# A Comparative Study of the Effects of Osaterone Acetate and Deslorelin Acetate on Sperm Kinematics and Morpho-Functional Parameters in Dogs

**DOI:** 10.3390/ani12121548

**Published:** 2022-06-15

**Authors:** Wojciech Niżański, Maria Eberhardt, Małgorzata Ochota, Christelle Fontaine, Xavier Levy, Joanna Pasikowska

**Affiliations:** 1 Department of Reproduction and Clinic of Farm Animals, Wrocław University of Environmental and Life Sciences, pl. Grunwaldzki 49, 50-366 Wrocław, Poland; maria.eberhardt@upwr.edu.pl (M.E.); malgorzata.ochota@upwr.edu.pl (M.O.); 2 Virbac Group, Global Marketing and Business Optimization Department, Companion Animals Veterinary Exclusive Ranges Section, 13ème Rue LID, 06511 Carros, France; christelle.speiser-fontaine@virbac.com; 3 Crecs, 58 BD Poumaderes, 32600 Isle Jourdain, France; vetlevy@gmail.com; 4 Veterinary Clinic, B. Krzywoustego Str. 105/22, 51-166 Wroclaw, Poland; j.h.pasikowska@gmail.com

**Keywords:** dog, semen quality, BPH, osaterone acetate, deslorelin acetate

## Abstract

**Simple Summary:**

Benign prostatic hyperplasia affects over 80% of old, non-castrated male dogs. In many cases, the severity of the symptoms means that treatment is required. The treatment of choice is surgical castration, which is unacceptable for the owners of stud dogs who are interested in therapy that does not exclude their animals from breeding. In those cases, treatment with deslorelin acetate or osaterone acetate offers an alternative to surgery. The aim of our study was to compare the changes in semen parameters over time. The deslorelin acetate implant gradually, during the course of its action, led to a full prevention the stud dogs from mating. However, in the first week following implant placement, in some individuals we observed an increase in sperm concentration (flare up effect), offering the possibility to obtain sperm in valuable dogs before the action of deslorelin acetate is completely established, precluding semen collection. On the other hand, osaterone acetate allowed for the continuous reproductive use of a stud dog, as it only affected some semen parameters, and mainly caused a temporary reduction in semen volume with the subsequent increase in semen concentration without altering the libido.

**Abstract:**

Benign prostatic hyperplasia (BPH) is one of the most common problems in older male dogs that often has a huge impact on their health and welfare. This article presents a comparison between osaterone acetate (Ypozane^®^; Virbac^®^)(OA) and deslorelin acetate (Suprelorin^®^; Virbac^®^)(DA), medications that are the main therapeutic alternative to castration in dogs with BPH. Forty dogs were divided into four groups: I—negative control (five dogs without BPH); II—positive control (10 individuals diagnosed with BPH); III—15 dogs treated with DA, and IV—10 individuals treated with OA. Semen fractions were collected on days 0 (day of treatment), 7, 14, and 21, and weeks 8, 12, 16, and 20. Macroscopic, microscopic and CASA analyses were performed. Both DA and OA significantly affected the properties of the canine ejaculate. The DA lead to the lack of libido and had lesser effects to the sperm function before it caused azoospermia, whereas OA had no effect on libido and only temporary reduction in seminal plasma volume was observed, which resulted in temporary deterioration in the percentage of motile and progressive spermatozoa.

## 1. Introduction

There is histological evidence of benign prostatic hyperplasia (BPH) in approximately 80% of dogs over 5 years of age [1,2,3]. Along with the development of BPH symptoms, there is deterioration in the patient’s general comfort and reproductive potential.

Previous studies have shown that BPH does not develop in dogs that are castrated, have a 5α-reductase type II deficiency [4,5], or have an underdeveloped pituitary gland [4], indicating that androgens are mainly responsible for prostate function as well as its excessive development. Due to this, surgical castration, which removes the main source of these hormones, is the treatment method of choice in dogs [6]. However, this is often unacceptable to owners of stud dogs who are particularly interested in solutions which do not preclude their animals from breeding. Hence, the recent interest in pharmacological treatments as an alternative for use in stud males, as well as for older patients with comorbidities and increased anesthetic risk.

In men, one of the drugs commonly used in BPH treatment is finasteride. This substance acts as inhibitor of 5α-reductase by blocking its enzymatic action. Finasteride effectiveness was also proved in dogs, reducing DHT level resulting in decreasing prostatic size and clinical signs related to BPH [7].

However, antiandrogens and GnRH receptor agonists seem to be the most frequently chosen drugs in current veterinary practice. The mechanism of action of antiandrogens is to block the androgen receptor (AR), preventing the binding of testosterone and dihydrotestosterone (DHT) and impairing the normal function of these hormones. The antiandrogen used for BPH treatment in dogs is osaterone acetate (OA). Its mechanism of action is competitive, blocking AR in the prostate, which blocks testosterone transport to the prostate and reduces the synthesis of androgen receptors, but it probably also decreases the activity of 5α-reductase [8].

On the other hand, long-acting GnRH analogues cause desensitisation of GnRH receptors (GnRH-R) and consequently a decrease in the circulating levels of LH and FSH. Then, after about four days of continuous use, GnRH analogues cause a marked suppression of reproductive function, called the down regulation effect [9,10]. Deslorelin acetate, in the form of a long-acting implant, is registered for use in male dogs to induce reversible loss of fertility. However, it has also been observed to induce a significant clinical improvement in BPH [10,11,12,13,14] by decreasing the peripheral blood testosterone level and consequently the DHT level in the prostate gland, resulting in an apparent reduction in its size during the course of treatment [10,11,12,13,15].

Deslorelin acetate and osaterone acetate have different mechanisms of action and other therapeutic applications. Implants containing deslorelin acetate are mainly used for reversible male castration, and while it is obvious that this drug should completely preclude a dog from reproducing, there is still a lack of detailed information about its influence on semen production and quality. In contrast, the mechanism of action of osaterone acetate, which is targeted at BPH therapy, would not be expected to influence semen quality. However, there is also a lack of detailed research on the ability of this drug to preserve reproductive activity during a course of treatment. In this article, we present the results of studies comparing the short and longer-term effects of osaterone acetate (Ypozane^®^; Virbac^®^) and deslorelin acetate (Suprelorin^®^; Virbac^®^) on sperm parameters and the overall reproductive capacity of male dogs with benign prostatic hyperplasia.

## 2. Materials and Methods

### 2.1. Animals and Study Groups

The research was approved by the II Local Ethical Commission for Animal Experiments at the University of Life Sciences in Wrocław (No 36/2014).

The experiment was carried out on forty non-castrated male dogs of different breeds and aged at least 5 years. Based on the results of the initial examination, which included clinical history taking, basic clinical examination, rectal examination, blood morphology, biochemistry and endocrine analysis, and B-mode US examination of the reproductive system—testes and prostate, patients were divided into two control groups (I and II) and two study groups (III and IV). Additionally, in dogs classified as groups II, III and IV, BPH was confirmed by US-guided fine needle biopsy (FNA). The slides were coded by the laboratory technician and sent for the national board certified veterinary clinical pathologist for evaluation.

#### 2.1.1. Group I (Negative Control—Healthy Stud Dogs)

According to published reports, the ejaculate parameters obtained in subsequent collections are only comparable in stud dogs in which semen was collected at regular intervals [16]. Thus, we decided to choose for our negative control group solely stud dogs of the previously proven ability to donate semen regularly, to ensure the repeatability and comparability of the obtained results.

The negative control group consisted of five healthy, non-castrated stud dogs of different breeds (two Welsh corgi cardigan, two Polish hounds, one Bedlington terrier, one pointer, one beagle, one Australian cattle dog and two mixed breed dogs), aged 5–10 years (average 6.5 years ±2 years; median 5.5 years) with weight from 8.7 to 28.3 kg (mean 17.08 kg (±6.21 kg); median 16.5 kg and no history or symptoms of reproductive tract disorders.

#### 2.1.2. Group II (Positive Control)

Ten non-castrated male dogs of different breeds (two Yorkshire terriers, two beagles, one Alaskan malamute, one Irish setter and four mixed breed dogs), aged 5–15 years (average 9.5 years ± 3.5 years; median 9.5 years) and weight ranged from 2.9 to 44 kg (mean 14.12 kg (±12.17 kg); median—12.65 kg) with BPH confirmed by US-guided FNA, whose owners decided to postpone treatment for a variety of reasons (e.g., working, hunting, valuable stud dogs)

#### 2.1.3. Group III (Deslorelin Acetate—SUPRELORIN^®^ VIRBAC^®^)

Fifteen non-castrated male dogs of different breeds (two boxers, one beagle, one dachshund, one leonberger, one miniature schnauzer, one Bernese mountain dog, one French shepherd (stud dog), one Labrador retriever, one American Staffordshire terrier, onemIrish setter and four mixed breed dogs), aged 6–15 years (mean 9.47 ± 2.13 years; median 10 years) with weight ranging from 7.6 kg to 46.5 kg (mean 27.6 kg (±14.65 kg), median—30 kg) and BPH confirmed by US-guided FNA.

#### 2.1.4. Group IV (Osaterone Acetate—YPOZANE^®^ VIRBAC^®^)

Ten non-castrated male dogs of different breeds (including four stud dogs) (two American Staffordshire terriers, one1 German shorthaired pointer, one Yorkshire terrier, 1 leonberger, one Airedale terrier, one German shepherd, one Irish setter, one Shetland sheepdog and one miniature schnauzer), aged 5–10 years (mean 7.6 years ±1.96 years; median 8 years) with weight ranging from 4.1 to 50.5 kg (mean 26.92 kg (±15.25 kg), median—27.35 kg) and BPH confirmed by US-guided FNA.

### 2.2. Study Design

A total of eight examinations were performed in each group on days 0 (D0), 7 (D7), 14 (D14), and 21 (D21) and in weeks 8 (W + 8), 12 (W + 12), 16 (W + 16), and 20 (W + 20). For each examination, an attempt was made to collect and evaluate the ejaculate.

#### 2.2.1. Drug Administration

##### Group III

On day 0 (D0), after the initial semen collection, patients were given 4.7 mg of deslorelin acetate (SUPRELORIN 4.7 mg implant for dogs; VIRBAC^®^ France). The implant was administered subcutaneously in the dorsal region between the lower neck and the lumbar region. As recommended by the manufacturer, each patient was given one implant, regardless of body weight.

##### Group IV

On day 0 (D0), after the first examination, patients started receiving the osaterone acetate (YPOZANE; VIRBAC^®^ France). The medication was administered per os at the manufacturer’s recommended dose: 0.25–0.5 mg/kg body weight every 24 h for 7 consecutive days.

### 2.3. Semen Analysis

Semen fractions were collected by manual stimulation into a plastic test tube. Fractions: the II- sperm rich and the III- prostatic fluid were used in the study [17]. In some individuals from group II (due to clinical BPH symptoms) and group III (due to the libido decreasing action of DA) the follow-up semen collections become impossible. In such cases, only descriptive statistics were used or statistical analysis performed only for individuals in which semen was collected at each of the following examinations.

#### 2.3.1. Assessment of II Fraction

Immediately after collection, the semen-rich fraction was submitted for macroscopic analysis to determine the volume. Motility was assessed subjectively by phase contrast microscopy (Nicon Eclipse E200; 400× zoom), using a 10 µL drop of ejaculate on a glass slide placed on a heated stage and covered with a cover slide [18]. Semen was then loaded into a 20 µm-deep Leja chamber for computer-assisted analysis of sperm concentration and motility performed using the HTM IVOS ver. 12.2 (Hamilton Thorne Biosciences, Beverly, MA, USA). The analysis was carried out using phase contrast optics at 1.89 × 10 zoom. Total sperm count in ejaculate (×10^6^), sperm concentration (×10^6^/^mL^), percentage of motile sperm (Motility—MOT, %), percentage of sperm with progressive motility (PMOT, %), and kinematic parameters: Curvilinear Velocity (VCL, µm/s), Average Path Velocity (VAP, µm/s), Straight Line Velocity (VSL, µm/s), Linearity (LIN,%), Straightness (STR,%), Amplitude of Lateral Head Displacement (ALH) µm), and Beat Cross Frequency (BCF, Hz), were assessed. The proportion of spermatozoa in the motility subcategories Rapid (VAP > 100 µm/s), Medium (9 µm/s < VAP < 100 µm/s), Slow (VAP < 9 µm/s) and Immotile, were also determined. Semen was evaluated by counting 200 motile sperm cells in each of five separate fields.

Morphological evaluation of spermatozoa was done by preparing a 10 μL smear of diluted semen on a microscope slide which was then stained using the Bydgoska method [19]. Slides were evaluated using a phase contrast microscope (Nikon E200, 1000×) and 200 spermatozoa were evaluated each time [20]. Similarly, the percentages of live and dead sperm were evaluated using the eosin-nigrosin method and counting up to 200 spermatozoa [18].

#### 2.3.2. Evaluation of the III Semen Fraction

Immediately after collection, the III semen fraction was submitted for macroscopic assessment to determine the volume and the presence of blood. Then, the III fraction was subjected to cytological evaluation using smears made from 10 μL of the prostate fractions. After drying, the slides were stained with Diff-Quick dye (Medion Diagnostic AG, Switzerland). Smears were evaluated using a phase contrast microscope (Nikon E200, ×400, ×600).

### 2.4. Statistical Analysis

Statistical analysis was performed using the STATISTICA 10.0 (StaSoft Inc., Kraków, Poland) program. The results for quantitative data are presented as mean, standard deviation (SD) and minimum and maximum values.

The Shapiro-Wilk test was used to test the null hypothesis [21]. In order to check the significance of the differences among groups and within each group, Student’s parametric *t* test and the U Mann-Whitney non-parametric tests were used.

The differences within groups in relation to D0 values (before drug administration) were compared. The comparison between control and studied groups were not made because of the naturally occurring physiological and individual discrepancies in the baseline results obtained in D0 in all studied groups [10,22].

In the control group II, only descriptive statistics were used due to problems with obtaining ejaculates, and thus obtaining only a small number of samples. In experimental group III, full statistical analysis was performed only for individuals in which semen was collected at each of the following examinations, as DA significantly decreased libido in some of the investigated dogs making semen collection impossible in the following examinations.

All statistical analyzes were performed at the significance level *p* = 0.05.

## 3. Results

### 3.1. Evaluation of II Semen Fraction

Neither a breed-dependent nor age-dependent relationship was observed. In healthy patients without prostate disease (Group I—negative control), semen was collected from each dog at each examination and no libido changes were observed. In contrast, the collection of semen from patients with clinical symptoms of BPH (Group II) was difficult, as most animals had poor libido and an aversion to ejaculation. Samples of semen in subsequent collections were obtained only from three individuals. In the DA group (Group III), semen was obtained initially from 11 individuals. Depending on the individual, it was possible to obtain semen either up to D7 (*n* = 1), D14 (*n* = 1), D21 (*n* = 7) or W + 8 (*n* = 2) after placement of the implant. Most patients (73%) maintained a copulative reflex until the end of the study, and approximately half of the individuals were also able to show partial penile erection. In 64% of individuals, owners observed an increase in libido in the first two weeks after implant placement. In the OA group (Group IV), semen was initially collected from six individuals. Semen samples were obtained during subsequent examinations, and no significant changes in libido were observed during the study.

In Group I, semen was assessed as normal (Table 1) and no significant changes were noted in the qualitative or quantitative parameters, or in sperm motility (Table 1 and Table 2).

In patients with clinical symptoms of BPH (Group II), the semen quality was variable depending on individual and the day of collection (Supplementary Materials, Tables S1 and S2), while in the DA group (Group III), semen was initially assessed as normal (Table 3). There were significant differences observed in the proportion of spermatozoa, with morphological defects starting from the 8th week of study (*p* = 0.03. Statistical analysis was performed only for samples collected at each of the following examinations) (Table 3). No significant changes in sperm motility were observed in group treated with DA as long as semen collection was possible (Table 4).

For Group IV, from week 8 a progressive decrease in the volume of the III fraction (*p* = 0.005) was observed (accompanied by a significant increase in sperm concentration, *p* = 0.05), a decrease in the percentage of motile sperm (*p* = 0.04) and the percentage of progressive sperm (*p* = 0.04). Starting from week 12, the volume began to rise again and did not differ significantly from the values before treatment (Table 5). During this therapy, no significant changes in sperm morphology or proportion of live spermatozoa were noted (Table 5). Most parameters describing sperm motility did not show significant differences during the study (Table 6). Only a significant increase in the percentage of immotile spermatozoa was observed on day 21 (D21) after the start of therapy (Table 6).

### 3.2. Evaluation of III Semen Fraction

**Figure 1:**
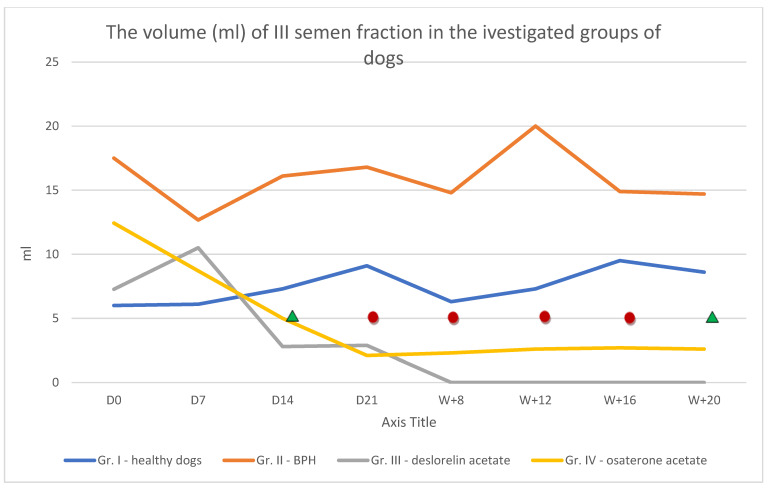
The volume of semen fraction III in Group I, II, III and IV of investigated dogs (mL) in subsequent examinations until week 20 *n* = 6; (mean; 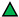 *p* < 0.05, 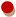 *p* < 0.01 in relation to D0).

Only samples from Group I had no blood found (either macroscopically or microscopically) on initial examination of fraction III. The presence of blood was noted in the samples from the other groups. The fraction III volume did not differ significantly in the negative control group throughout the study and ranged from 6 mL to 9.5 mL (Figure 1). In patients from Group II, collection of the III fraction was difficult, as it was for the II fraction, and the mean volume of this fraction ranged from 12.67 to 20 mL. In Group III, the mean volume of fraction III initially increased to 10.5 mL on day 7 after implant placement, and then decreased gradually to 2.89 mL. Day 21 was the last time that semen fraction III was obtained from all individuals (Figure 1) in Group III. In Group IV, the volume decreased starting from day 7 (D7) of therapy until week 20 (W + 20), when the volume began to increase again (Figure 1)

## 4. Discussion

Semen quality is of great importance in stud dogs, but this can be affected by age-related prostate disorders. Therefore, an effective prostate therapy that supports the maintenance of fertility is sought. This clinical study for the first time compares the effect of two substances commonly used in BPH therapy in dogs, osaterone acetate (OA) and deslorelin acetate (DA), on semen and sperm quality, and thus on the possibility of producing offspring.

Factors affecting semen quality in dogs are the age and general health of the male, together with the frequency of collection, the presence of a bitch in oestrus and the season of the year [23,24]. Dog ejaculates have intra-individual and inter-individual differences in quality and volume [23,24]. The quantitative parameters of the ejaculate (mainly the total number of spermatozoa) obtained during subsequent collections are only comparable when sperm was collected at regular intervals, e.g., every 7 days [23]. Therefore, we decided to choose individuals for our negative control group among stud dogs, to ensure repeatable ejaculate characteristics. As reported, prostate disorders i.e., hypertrophy or inflammation, may impair secondary semen characteristics [24]. Similarly, in our research we observed reduced semen collection in dogs from Control Group II (nontreated dogs with confirmed BPH) and low semen quality compared to that of healthy dogs from Group I.

The main differences between Groups III and IV were changes in libido and the ability to ejaculate. In dogs treated with desloreline acetate, we were able to collect semen up to day 21 after placing the implant. This was similar to the results published by Junaidi et al. [14,15], Trigg et al. [13], and Polisca et al. [22]. In contrast, Romagnoli et al. [25] were able to collect semen on average for the first 54 days, and in one individual the collection was still possible at day 80 after implant placement. Based on this, it can be assumed that the impact of desloreline acetate on semen donation is individual-dependent and can occur from 21 to 80 days after implant placement. Hence, the owners of stud dogs should be advised to isolate males for at least one month after implant placement to avoid any unplanned mating. However, it should be remembered that in individual cases fertility could be maintained for almost three months [25].

It is worth noting that, as reported by their owners, most dogs from Group III (DA group) had a temporary increase in libido within the first two weeks after implant placement, suggesting the appearance of a flare-up effect. This may be the result from a temporary increase in testosterone bloodstream level and, in consequence, intensified testosterone-dependent behaviour [26]. Thereafter, 50% of males showed only a partial erection and 75% showed copulatory movements. Similar behaviour was reported by Junaidi et al. [14]. Many authors have reported observing a gradual decrease in some quantitative or qualitative semen parameters from around day 14 after implant placement [12,13,14,21,27]. Junaidi [14] reported a reduction in ejaculate volume followed by decreased sperm concentration and motility [14]. However, Romagnoli et al. [25] noted an increase in sperm motility in some individuals immediately after implant placement. Similarly, in our research, some individuals had an increase in the sperm concentration in the first week after implant placement. This was most likely associated with the previously described flare-up effect of the GnRH agonists [10]. From a clinical point of view, it is worth noting that this flare-up effect creates the opportunity to collect and preserve semen before it becomes impossible due to the full clinical effect of DA. Furthermore, this approach provides a chance for a temporal improvement of the ejaculates collected from dogs with diagnosed reduced sperm quality. Both outcomes might be important to augment the reproductive potential of valuable stud dogs and have practical value for stud dog owners.

In dogs treated with deslorelin acetate, we observed an increase of secondary morphological defects in spermatozoa starting from W + 2 of the study, which became significant from W + 8. However, no primary defects were noted, which could indicate undisturbed spermatogenesis in these dogs. Similar changes have been reported in previous studies [12,13,14]. In contrast, Romagnoli et al. (2012) and Ludwig et al. (2009) did not observe a significant influence of GnRH analogues on sperm morphology, as this chemical does not affect testicular tissue. Hence, during DA therapy mostly secondary defects would be observed, i.e., a tendency to decline in overall sperm motility, progressive motility and rapid motility, and an increase of static spermatozoa were noted. In our observations, all these differences were noted, but were not significant when compared to D0 values, which is similar to the results published previously [12,13,14], demonstrating that with GnRH analogues, sperm function is preserved for as long as it is possible to obtain an ejaculate. It is most probable that the pituitary-gonadal axis is slowly silenced by the GnRH receptor agonists, and that the spermatogenic process is gradually inhibited. These results were confirmed by the observations of histological changes in the testes during deslorelin acetate therapy reported by Junaidi et al. [15], who showed the progressive atrophy of seminiferous tubules. Atrophy was seen in 89.8% of tubules assessed on day 41 of therapy, and in 99.8% of tubules assessed on day 101, and spermatogenesis was arrested at the spermatogonial stage [14]. It was suggested that sperm reserves are gradually released with ejaculation during the weeks following deslorelin acetate therapy. Hence, in the later sperm collections, the higher percentage of secondary defects, and the tendency for reduced sperm motility, could be due to a prolonged residence time of the gametes in the epididymis, and the lower sperm concentration, due to the gradual depletion of the reserves. This clearly explains that the initial increase is most likely associated with the flare-up effect, followed by a decrease in the volume of semen fraction III. In our study, the latest we were able to collect the III fractions was day 21 after implant placement, and the inability to obtain the III fraction persisted until the end of the experiment.

No significant effect on libido was observed in Group IV. Our findings correspond with a previous publication [27] reporting no problems with obtaining semen from dogs throughout the OA therapy period. The cited paper also showed that osaterone acetate (at doses of either 0.2 mg/kg or 0.5 mg/kg) did not significantly affect semen volume, concentration, motility or sperm vitality. On the contrary, in our experiment, alterations in the volume of ejaculates were observed. Initially, until week 8 of the study, a significant decrease was noted in the volume of the II semen fraction (1 mL vs. 2.5 mL), followed by a gradual increase. It can be speculated that the significant decrease in motility observed during this period might be associated with the higher concentration of gametes caused by a transient decrease in the amount of secretion produced by the prostatic parenchyma, i.e., seminal plasma. A decrease in the volume of semen fraction III and an increase in sperm concentration during administration of osaterone acetate was evident.

Tsustui et al. [27] reported an increase in the percentage of spermatozoa with morphological defects, specifically sperm tail defects, during therapy with osaterone acetate, increasing from about 5% initially to 10% after four weeks of treatment, remaining at this level for the next 6 weeks, before returning to 5%. However, we did not find any significant differences in sperm morphology during osaterone acetate administration, confirming previous reports that it did not affect spermatogenesis or sperm maturation [28]. However, we observed a significant reduction in the volume of semen fraction III with increased total spermatozoa concentration in the osaterone acetate group. This had decreased by half on day 7 of OA administration, and the lowest volumes were collected on day 21 of therapy. Whether or not the lower III fraction volume had an immediate effect on the changes in semen motility would need further study but considering the observed higher semen concentration in the OA group it seems to be the most obvious interpretation.

## 5. Conclusions

Our study showed that both deslorelin acetate and osaterone acetate significantly affected the properties of canine ejaculate. The use of deslorelin acetate excluded stud dogs from reproduction and made it impossible to collect sperm from about a month after the implant placement, although sperm function seemed to be preserved until ejaculate collection became impossible. Moreover, DA increased only secondary morphological defects in spermatozoa without affecting spermatogenesis, as no primary defects were noted. On the other hand, in males treated with osaterone acetate, the therapy had no effect on libido and the patients’ ability to ejaculate but led to a temporary reduction in seminal plasma volume and increased semen concentration. OA also led to a temporary deterioration in the percentage of motile and progressive spermatozoa without changing the rest of the investigated sperm parameters, which might be associated with the higher concentration of gametes caused by a transient decrease in seminal plasma volume.

## Figures and Tables

**Table 1:** Values of qualitative and quantitative parameters of the II sperm fraction samples collected from dogs in control group I (negative control- healthy stud dogs) compared to the results from day 0 (*n* = 5). Results are presented as mean ± SD.

	II Fraction Volume (mL)	Concentration (×10^6^/mL)	Total Sperm Count (×10^6^)	Motility (%)	Progressive Motility (%)	Defects (%)	Live Sperm (%)
D0 *n* = 5	2.1 ± 0.5	220.8 ± 163.8	432.2 ± 268.3	96.8 ± 2.2	70.4 ± 10.2	9.2 ± 3.6	95.3 ± 4.8
D7 *n* = 5	1.9 ± 0.1	217.4 ± 154.1	410.3 ± 327.8	97.0 ± 2.9	75.0 ± 3.2	7.6 ± 4.2	97.0 ± 1.1
D14 *n* = 5	2.1 ± 1.0	266.8 ± 174.3	590.8 ± 549.1	95.6 ± 3.3	57.0 ± 31.3	10.2 ± 7.2	97.3 ± 2.3
D21 *n* = 5	2.0 ± 0.7	293.5 ± 194.5	507.6 ± 179.0	90.0 ± 1.3	73.7 ± 5.2	10.1 ± 3.1	98.1 ± 1.1
W + 8 *n* = 5	1.7 ± 0.5	285.3 ± 303.8	397.0 ± 400.8	93.5 ± 8.4	58.7 ± 24.3	11.7 ± 4.3	98.5 ± 0.6
W + 12 n=5	2.1 ± 1.0	229.2 ± 133.1	412.8 ± 243.1	94.5 ± 5.8	66.5 ± 6.5	7.5 ± 2.7	98.0 ± 1.4
W + 16 *n* = 5	1.6 ± 0.8	452.0 ± 291.4	613.9 ± 420.4	97.5 ± 1.3	73.8 ± 7.8	7.5 ± 1.1	96.3 ± 2.1
W + 20 *n* = 5	1.2 ± 1.0	275.1 ± 183.0	387.7 ± 485.5	96.5 ± 3.1	57.3 ± 18.2	8.5 ± 3.1	98.6 ± 0.3

**Table 2:** Characteristics of sperm motility in the II sperm fraction samples collected from dogs in control group I (negative control- healthy stud dogs) compared to the results from day 0 (*n* = 5). Results are presented as mean ± SD.

	VAP	VSL	VCL	ALH	BCF	STR	LIN	RAPID	STATIC
D0 *n* = 5	146.4 ± 21.1	138.6 ± 31.6	187.2 ± 38.9	5.7 ± 1.3	33.3 ± 5.0	92 ± 2.8	73 ± 6.3	77.4 ± 13.5	3.2 ± 2.2
D7 *n* = 5	170.9 ± 9.5	155.0 ± 3.5	226.6 ± 30.8	6.9 ± 1.1	34.3 ± 4.2	90.3 ± 4.5	70 ± 8.9	85 ± 7.6	2.65 ± 2.5
D14 *n* = 5	147.4 ± 45.3	149.9 ± 7.7	193.7 ± 5	6.2 ± 1.2	29.8 ± 4.6	85 ± 12.1	66 ± 16.3	66 ± 35.5	4.4 ± 3.3
D21 *n* = 5	158.0 ± 14.0	142.2 ± 10.7	206.5 ± 3	6.2 ± 1.1	34.0 ± 2.6	89 ± 3.2	67 ± 5.4	84.83 ± 5.0	2 ± 1.3
W + 8 *n* = 5	141.8 ± 31.3	130.0 ± 28.7	188.3 ± 27.1	6.2 ± 1.5	33.6 ± 6.0	91 ± 3.8	68 ± 10.9	66.17 ± 7.4	6.5 ± 8.4
W + 12 *n* = 5	148.1 ± 19.1	133.7 ± 11.4	195.7 ± 33.8	6.3 ± 1.4	31.3 ± 7.9	90 ± 5.4	69 ± 7.8	75.5 ± 9.88	5.5 ± 5.8
W + 16 *n* = 5	169.9 ± 14.3	153.1 ± 14.3	216.7 ± 19.6	6.2 ± 0.9	31.5 ± 4.0	89 ± 4.9	71 ± 7.0	85.3 ± 2.6	2.5 ± 1.3
W + 20 *n* = 5	138.6 ± 31.6	123.7 ± 34.1	191.6 ± 3	7.7 ± 1.6	29.2 ± 5.0	87.3 ± 5.6	64.3 ± 8.8	64.8 ± 17.7	3.5 ± 3.1

**Table 3:** Values of qualitative and quantitative parameters of the sperm II fraction samples collected from dogs in Group III (BPH treated with Deslorelin Acetate—SUPRELORIN^®^ VIRBAC^®^) compared to the study on day 0 (*n* = 11). Results presented as mean ± SD (^A^ *p* < 0.05) *.

	II Fraction Volume (mL)	Concentration (×10^6^/mL)	Total sperm Count (×10^6^)	Motility (%)	Progressive Motility (%)	Defects (%)	Live Sperm (%)
D0 *n* = 11	2.2 ± 1.2	167.6 ± 148.8	433.2 ± 532.3	87.6 ± 8.2	61.6 ± 18.6	19.9 ± 10.1	94.6 ± 3.9
D7 *n* = 10	2.6 ± 1.5	215.2 ± 163.8	534.4 ± 401.2	82.6 ± 21.4	53.8 ± 25.1	19.1 ± 5.6	91.5 ± 5.1
D14 *n* = 9	2.5 ± 1.7	204.7 ± 155.6	406.3 ± 282.8	72.6 ± 34.4	50.7 ± 27.5	29.3 ± 8.2	91.1 ± 3.7
D21 *n* = 2	2.4 ± 1.0	172.4 ± 119.9	392.8 ± 331.9	89.4 ± 10.0	63.6 ± 19.6	24.1 ± 10.9	91.6 ± 5.1
W + 8 *n* = 2	2.9 ± 1.6	97.3 ± 128.6	182.2 ± 221.5	65 ± 38.2	35.5 ± 23.3	48.5 ^A^ ± 6.4	86.5 ± 8.5

* Full statistical analysis was performed only for individuals in which semen was collected at each of the following examinations.

**Table 4:** Characteristics of sperm motility in semen fraction II samples collected from dogs in Group III (BPH treated with Deslorelin Acetate—SUPRELORIN^®^ VIRBAC^®^) compared to the study from day 0 (*n* = 11). Results are presented as mean ± SD.

	VAP	VSL	VCL	ALH	BCF	STR	LIN	RAPID	STATIC
D0 *n* = 11	150.5 ± 22.6	139.9 ± 22.4	196.0 ± 31.8	6.5 ± 1.6	31.0 ± 5.0	92 ± 4.3	72 ± 10.0	663 ± 20.5	12.36 ± 8.19
D7 *n* = 10	145.8 ± 36.2	130.5 ± 32.8	196.2 ± 45.4	6.9 ± 2.0	29.9 ± 4.3	89 ± 6.0	67 ± 11.6	61.8 ± 27.2	17.4 ± 21.14
D14 *n* = 9	141.3 ± 31.4	128.3 ± 28.8	197.5 ± 41.0	6.43 ± 2.2	31.3 ± 5.7	90 ± 3.6	67 ± 11.8	56.1 ± 31.1	27.43 ± 34.41
D21 *n* = 2	155.9 ± 21.4	143.8 ± 24.0	207.5 ± 19.2	6.7 ± 1.8	31.2 ± 4.0	91 ± 3.9	70 ± 12.1	69.2 ± 21.0	10.56 ± 10.03
W + 8 *n* = 2	129.2 ± 10.3	117 ± 22.8	187.3 ± 43.6	6.4 ± 3.0	28.3 ± 1.7	89 ± 9.9	66 ± 24.8	42.5 ± 33.2	35.0 ± 38.2

**Table 5:** Values of qualitative and quantitative parameters of the sperm fraction II samples collected from dogs in Group IV (BPH treated with Osaterone Acetate—YPOZANE^®^ VIRBAC^®^)—comparison in relation to the study on day 0 (*n* = 6). Results presented as mean ± SD (^A^ *p* < 0.05; ^B^ *p* < 0.01).

	II Fraction Volume (mL)	Concentration (×10^6^/mL)	Total Sperm Count (×10^6^)	Motility (%)	Progressive Motility (%)	Defects (%)	Live Sperm (%)
D0 *n* = 6	2.5 ± 0.8	287.4 ± 178.9	678.6 ± 384.9	90.3 ± 7.9	50.7 ± 23.7	35.7 ± 24.5	95.6 ± 2.9
D7 *n* = 6	1.7 ± 0.5	238.2 ± 272.5	269.9 ± 215.2	87.3 ± 8.02	39.3 ± 20.1	41.3 ± 23.1	94.0 ± 0.7
D14 *n* = 6	1.6 ± 1.0	385.4 ± 187.0	623.4 ± 363.0	83 ± 13.2	38.6 ± 26.0	32.3 ± 23.9	93.0 ± 3.6
D21 *n* = 6	1.4 ± 0.9	739.0 ^A^ ± 558.0	772.6 ± 569.9	68.6 ^A^ ± 20.9	21.6 ^A^ ± 16.4	31.9 ± 19.5	94.3 ± 0.9
T + 8 *n* = 6	1.0 ^B^ ± 0.6	520.2 ^A^ ± 181.7	501.6 ± 377.5	84.8 ± 7.8	37.7 ± 20.7	37.2 ± 18.1	93.9 ± 4.2
W + 12 *n* = 6	1.6 ± 0.4	350.8 ± 88.7	524.0 ± 35.3	84.3 ± 13.2	44.5 ± 17.7	27.8 ± 12.8	93.3 ± 3.8

**Table 6:** Characteristic of sperm motility in semen fraction II samples collected from dogs in Group IV (BPH treated with Osaterone Acetate—YPOZANE^®^ VIRBAC^®^)- comparison in relation to the study on day 0 (*n* = 6). Results presented as mean ± SD (^A^ *p* < 0.05).

	VAP	VSL	VCL	ALH	BCF	STR	LIN	RAPID	STATIC
D0 *n* = 6	124.7 ± 32.0	115.0 ± 28.7	159.6 ± 48.2	6.0 ± 0.7	43.1 ± 36.5	94 ± 10.7	76 ± 20.1	56.2 ± 26.7	9.7 ± 7.9
D7 *n* = 6	99.1 ± 32.4	87.7 ± 33.3	130.8 ± 25.7	5.68 ± 0.6	25.4 ± 9.3	85 ± 11.2	64 ± 16.2	46.3 ± 15.9	11.3 ± 7.0
D14 *n* = 6	107.0 ± 52.3	95.6 ± 48.2	152.2 ± 68.2	6.1 ± 1.2	30.6 ± 5.2	87.4 ± 3.6	62 ± 9.2	42.8 ± 23.0	25.3 ± 18.2
D21 *n* = 6	101.9 ± 21.9	87.6 ± 25.9	154.7 ± 22.3	6.9 ± 2.0	25.7 ± 5.9	84 ± 8.5	57 ± 16.3	26.0 ± 16.2	31.4 ^A^ ± 20.9
W + 8 *n* = 6	112.9 ± 14.8	98.8 ± 17.3	172.1 ± 37.1	6.5 ± 1.6	30.1 ± 5.7	86 ± 7.9	59 ± 13.8	43.5 ± 21.5	15.2 ± 7.8
W + 12 *n* = 6	121.6 ± 17.7	112.5 ± 17.0	165.7 ± 21.5	5.9 ± 1.1	33.2 ± 3.7	91 ± 1.0	69 ± 4.8	52.3 ± 12.3	9.3 ± 7.9
W + 16 *n* = 6	129.6 ± 20.1	113.9 ± 17.8	181.8 ± 38.4	6.2 ± 1.7	31.4 ± 3.2	86 ± 4.0	63 ± 11.0	60.7 ± 23.0	6.0 ± 3.6
W + 20 *n* = 6	111.5 ± 35.1	98.6 ± 35.2	160.7 ± 20.9	6.4 ± 2.2	30.4 ± 0.9	86 ± 7.1	61 ± 16.4	41.6 ± 32.0	22.4 ± 19.6

## Data Availability

The data that support the findings of this study are available from the corresponding author [WN], upon reasonable request.

## Supplementary Materials

The following are available online at https://www.mdpi.com/article/10.3390/ani12121548/s1, Figure S1: title, Table S1: title, Video S1: title. Table S1: Values of qualitative and quantitative parameters of the II sperm fraction samples collected from dogs in control group II (positive control- untreated animals with BPH) (n = 3*). Results presented as mean ± SD, Table S2: Characteristic of sperm motility in the II sperm fraction samples collected from dogs in control group II (positive control- untreated animals with BPH) (n = 3, due to BPH related problems in obtaining semen in some individuals). Results presented as mean ± SD*.
