# Supplementary material for: A Comparative Study of the Effects of Osaterone Acetate and Deslorelin Acetate on Sperm Kinematics and Morpho-Functional Parameters in Dogs
Source: Animals (Basel). 2022 Jun 15;12(12):1548. doi: 10.3390/ani12121548 (PMC9219488; doi:10.3390/ani12121548)
Supplement: Supplementary file 1 [file animals-12-01548-s001.zip › animals-1752802-supplementary.pdf]

Supplementary Materials

Table S1. Values of qualitative and quantitative parameters of the II sperm fraction samples collected from dogs in control group II (positive control- untreated animals with BPH) (n = 3*). Results presented as mean ± SD

	**II fraction volume (ml)**	**Concentration (x10^6/ml)**	**Total sperm count (x10^6)**	**Motility (%)**	**Progressive motility (%)**	**Defects (%)**	**Live sperm (%)**
**D0** n=3	2.4 ±1.3	404.3 ±19.5	969.2 ±473.5	97.7 ±0.6	55.3 ±17.2	18.5 ±1.3	98 ±0
**D7** n=3	2.8 ±0.9	121.5 ±95.6	396.9 ±401.2	92 ±9.54	55.3 ±11.7	12.2 ±7.4	97.7 ±1.3
**D14** n=3	2.6 ±1.4	175.5 ±100.7	361.0 ±61.6	92.3 ±4.5	54.67 ±30.9	20.7 ±13.0	97.2 ±1.8
**D21** n=3	1.9 ±1.3	19.3 ±14.5	45.8 ±52.0	62 ±4.2	47 ±7.1	12 ±2.1	99.5 ±0
**W+8** n=3	1.2 ±1.2	71.5 ±27.6	109.5 ±90.4	93.5 ±5.0	51 ±25.5	36.4 ±3.9	95.75 ±3.9
**W+12** n=3	1.4 ±0.9	118.5 ±144.5	227.4 ±303	73 ±21.2	3 ±4.2	25 ±1.4	89 ±7.1
**W+16** n=3	2.5 ±0	209.0 ±101.1	522.5 ±252.7	95.5 ±2.1	67 ±14.14	29.5 ±22.6	96.5 ±0.7
**W+20** n=3	1.7 ±0.8	191.3 ±203.4	426.5 ±515.4	82 ±16.5	55.7 ±19.7	24.7 ±7.3	95.3 ±2.3

** only the descriptive statistics were used due to problems with obtaining ejaculates, and thus a small number of samples*

Table S2. Characteristic of sperm motility in the II sperm fraction samples collected from dogs in control group II (positive control- untreated animals with BPH) (n = 3, due to BPH related problems in obtaining semen in some individuals). Results presented as mean ± SD*

	**VAP**	**VSL**	**VCL**	**ALH**	**BCF**	**STR**	**LIN**	**RAPID**	**STATIC**
**D0 n=3**	138.4 ±28.3	119.4 ±24.3	196.4 ±29.7	6.5 ±1.1	30.9 ±1.5	85 ±2	61 ±5.7	67.7 ±23.3	2.33 ±0.6
**D7 n=3**	151.3 ±32.0	133.1 ±29.4	231.7 ±51.4	8.4 ±2.5	30.5 ±2.3	87 ±1.5	58. ±4.2	63 ±14.2	8 ±9.5
**D14 n=3**	135.1 ±53.5	117.2 ±50.7	195.8 ±66.6	7.5 ±1.4	27.3 ±6.6	85 ±8.1	59 ±14.5	61 ±34.9	7.7 ±4.5
**D21 n=3**	167.4 ±4.0	159.4 ±1.4	219.7 ±17.5	7.2 ±1.6	33.8 ±6.7	94 ±0	72 ±2.8	49 ±7.1	38 ±4.2
**W+8 n=3**	134.3 ±31.3	118.6 ±33.2	198.5 ±4.4	8.0 ±3.0	32.1 ±1.5	86 ±4.2	50 ±31.8	58 ±29.7	6.5 ±5.0
**W+12 n=3**	69.4 ±23.5	61.7 ±26.4	110.3 ±15.3	2.5 ±3.5	27.8 ±14.4	86 ±6.4	54 ±14.1	3 ±4.2	27 ±21.2
**W+16 n=3**	159.7 ±6.5	145.6 ±9.2	211 ±5.4	6.9 ±0.7	34.0 ±0.8	90 ±2.8	69 ±7.1	74.5 ±4.5	4.5 ±2.1
**W+20 n=3**	138.0 ±31.6	125.8 ±28.6	200.0 ±37.2	7.3 ±0.6	30.6 ±7.1	91 ±1.5	63 ±2.5	60.3 ±22.9	18 ±16.5
